# Clinical outcomes of drug-coated balloon vs. second-generation drug-eluting stent for coronary in-stent restenosis

**DOI:** 10.1007/s00392-025-02781-7

**Published:** 2025-11-25

**Authors:** J. Krefting, N. Krüger, C. Friess, C. Gräßer, R. Schmieder, F. Starnecker, M. Schwab, S. Kufner, T. Trenkwalder, F. Voll, H. B. Sager, T. Kessler, E. Xhepa, F. Offenborn, T. Dreischulte, D. Giacoppo, S. Cassese, A. Kastrati, H. Schunkert, M. von Scheidt, J. Wiebe

**Affiliations:** 1 Department of Cardiology, German Heart Center Munich, Technical University of Munich, Munich, Germany; 2 German Center for Cardiovascular Research E.V. (DZHK), Partner Site Munich Heart Alliance, Munich, Germany; 3 Allgemeine Ortskrankenkasse (AOK) Bayern, Munich, Germany; 4 Institute of General Practice and Family Medicine, LMU University Hospital, LMU Munich, Munich, Germany

**Keywords:** In-, Stent restenosis, Drug, Coated balloon, Second, Generation drug, Eluting stent, Real, World evidence

## Abstract

**Background:**

Current guideline recommendations for the treatment of coronary in-stent restenosis (ISR) are primarily based on evidence from smaller, randomized trials comparing drug-coated balloons (DCBs) and second-generation drug-eluting stents (DES).

**Purpose:**

The purpose was to compare clinical outcomes between DCB and DES in routine clinical practice.

**Methods:**

In this population-based cohort study, we identified 10,292 patients with ISR who underwent either DCB or DES intervention. After 1:1 propensity score matching, 3942 patients treated with either DCB or second-generation DES were included in the analysis. The composite primary end point was all-cause mortality or myocardial infarction (MI) at 1 year. Secondary end points included each component of the primary end point. The safety end point was defined by hospitalization due to bleeding events.

**Results:**

At 1-year follow-up, the primary outcome occurred in 10.4% (*n* = 206) of the DCB group versus 12.9% (*n* = 254) of the DES group (HR, 0.77; 95% CI, 0.64–0.93). Among secondary outcomes, all-cause mortality was lower in the DCB group (6.3%, *n* = 125) compared to the DES group (8.1%, *n* = 160; HR, 0.75; 95% CI, 0.59–0.94), as was hospitalization for bleeding (2.5%, *n* = 50 vs. 4.2%, *n* = 82; HR, 0.65; 95% CI, 0.45–0.96). Rates of MI were similar between groups (HR, 0.83; 95% CI, 0.63–1.10).

**Conclusion:**

DCBs were associated with lower rates of the primary end point all-cause death or myocardial infarction, as well as secondary end points all-cause death and bleeding compared with second-generation DES. These findings suggest that DCBs may represent a safe and effective alternative to DES in selected patient populations.

**Graphical Abstract:**

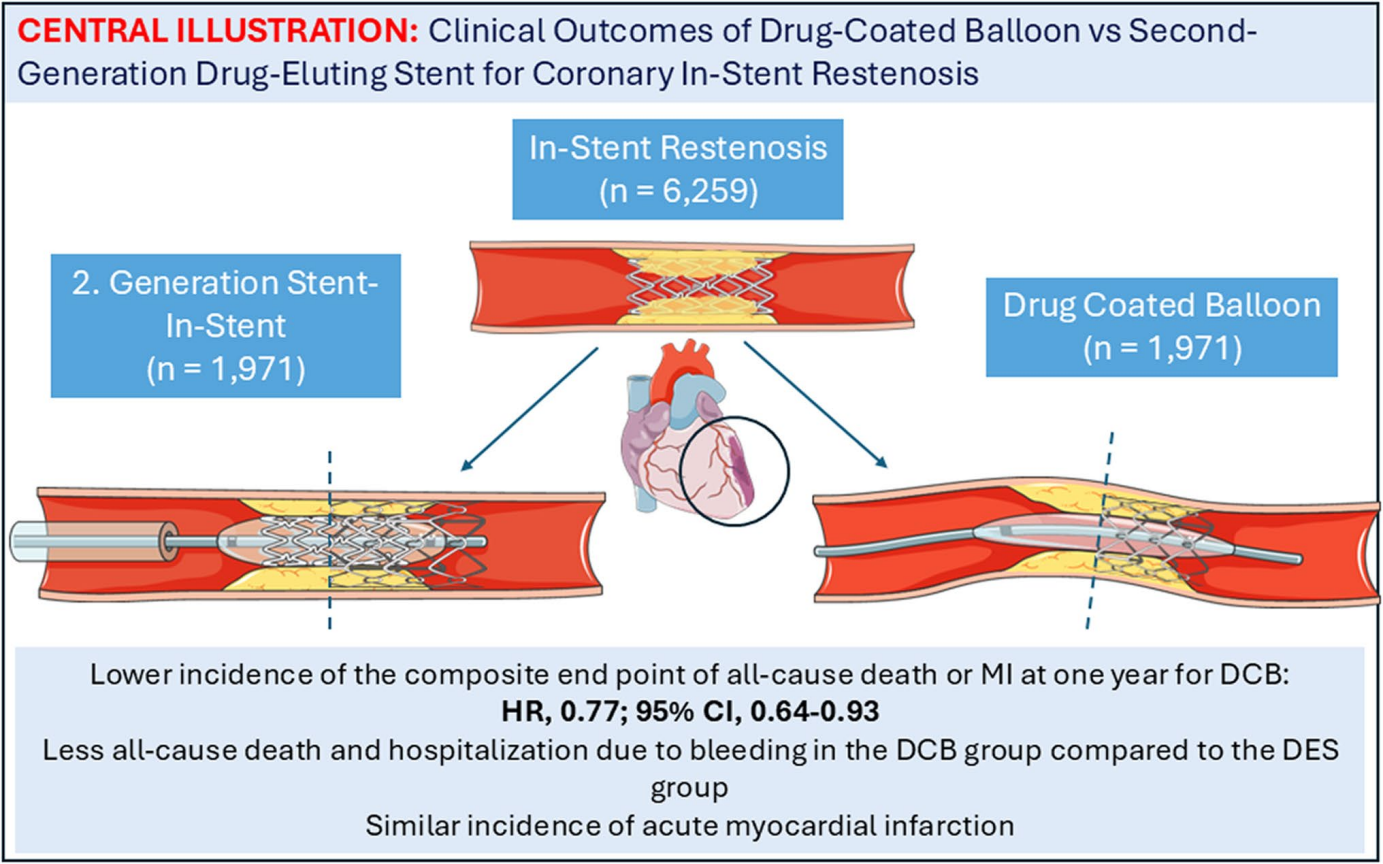

**Supplementary Information:**

The online version contains supplementary material available at 10.1007/s00392-025-02781-7.

## Introduction

Coronary in-stent restenosis (ISR) is a major limitation of percutaneous coronary intervention (PCI) and an important challenge for interventional cardiology.[1–3] Despite medical and technological advances, ISR continues to affect 5%–10% of contemporary PCI cases and remains a major cause of procedure failure.[4] While former guidelines recommended the use of either drug-eluting stents (DES) or drug-coated balloons (DCB) for the treatment of ISR with Class I indication[3], recent updates to the ESC guidelines now favor the use of DES over DCB for ISR due to higher target-lesion revascularization (TLR) in patients treated with DCB.[5, 6]

Small trials including PEPCAD-DES,[7] ISAR-DESIRE 3,[8] and the recent AGENT IDE study[9] demonstrated the superiority of DCB over plain balloon angioplasty, leading to the Food and Drug Administration’s approval of a paclitaxel-coated balloon for the treatment of coronary disease in March 2024.[10]

Investigations into the comparative safety and efficacy of DCB versus first-generation DES highlighted the noninferiority of DCB to first-generation DES[8] and even showed a numerically lower number of all-cause deaths.[11, 12]

These findings prompted further investigations comparing DCBs with second-generation DES, showing a reduced need for repeat TLR after the use of second-generation DES, as evidenced by studies such as RIBS IV.[13] However, concerns about long-term outcomes due to multimetal layers and reduced vasomotion from repeated stenting remain. Direct comparisons between DCB and second-generation DES for recurrent ISR have shown comparable outcomes.[14]

Despite these developments, most comparative data originate from smaller randomized trials with limited generalizability, leaving uncertainty about the relative safety and effectiveness of DCBs and second-generation DES in routine clinical practice. To address this gap,[15] we conducted a large, propensity score–matched cohort study to compare 1-year clinical outcomes of patients with ISR treated with either DCB or second-generation DES in patients routinely seen in clinical practice.

## Methods

### Data sources and implementation process

This study used German health insurance claims data from the Observational Bavarian Health Insurance Registry (OBSERVABLE).[16] This database is a comprehensive repository of secondary health data sourced from the Allgemeine Ortskrankenkasse (AOK) Bayern. The data include detailed information for over 2.3 million individuals across Bavaria aged 18 years and older who were diagnosed with an atherosclerotic disease between January 1, 2012 and December 31, 2021. The database includes anonymized patient-level information on demographics, diagnoses, medical procedures, drug prescription fills from pharmacies, and vital status (Supplemental Table 1). The data encompass all levels of care, from inpatients to outpatient services, reflecting the broad spectrum of healthcare utilization seen in cardiovascular medicine, making it a fit-for-purpose dataset for this analysis.

### Study design

To evaluate the safety and effectiveness of DCB vs. second-generation DES for the treatment of ISR, we employed a population-based cohort study design. Patients with a recorded instance of ISR who were treated with PCI within 3 days were selected into the cohort. This approach allowed the capture of acute treatment decisions and outcomes in routine care settings. Individuals were followed from the date of intervention to observed outcomes or a maximum of 1 year.

### Population

Inclusion required a recorded ICD-10 code specific to ISR (I25.16), paired with a record of a procedure code specific to a revascularization procedure within three days of the diagnostic code entry. Patients treated with bare-metal stents (BMS), first-generation DES, or plain balloon angioplasty (POBA) were subsequently excluded. In a secondary benchmarking cohort, patients treated with POBA were retained to enable direct comparison with contemporary DES and DCB.

To ensure adequate assessment of baseline covariates, only individuals with a minimum of 12 months of continuous enrollment in the database prior to the index revascularization procedure were included. The analysis was therefore restricted to patients identified between January 1, 2013 and December 31, 2021.

### Outcomes

The primary composite end point was defined as a composite of all-cause mortality or myocardial infarction (MI). Secondary end points included the individual components of the primary composite end point. MI was defined as ICD-10 codes I21 and I22, and a subsequent revascularization (PCI or CABG) procedure was required for validation, as this approach has shown similar results to randomized trials when emulating such.[17] The safety end point was defined by hospitalization due to bleeding events. ICD-10 codes used for bleeding are provided in Supplemental Table 1. To assess robustness, we conducted sensitivity analyses: (1) restricting to cases of single-vessel PCI at the time of ISR treatment and (2) restricting to patients with single-vessel coronary artery disease (CAD). The latter increases the likelihood that subsequent reinterventions reflect target lesion failure. Additionally, to evaluate residual confounding after propensity score matching, incident pneumonia and total hip replacement were assessed as negative control outcomes to ensure the validity of our results.

The comparison of antiplatelet therapy between the two groups was conducted by examining pharmacy dispensing of P2Y12 inhibitors after the ISR procedure. Utilizing both Anatomical Therapeutic Chemical (ATC) and pharmaceutical central number (PZN) codes allowed for precise calculation of daily doses available to each patient. Metrics were calculated for all reimbursements. To prevent distortion of the analysis, the number of P2Y12 daily doses were limited to a maximum of 400 per patient per year.

### Ethical considerations and study conduct

This study was conducted in accordance with the internationally accepted recommendations for clinical investigation as outlined in the Declaration of Helsinki of the World Medical Association. Ethics approval was obtained from the Ethics Committee of the Technical University Munich, as documented under the approval number 2019–50-S-SR.

### Statistical analysis

To adjust for potential confounding between the DCB and DES treatment, we employed a propensity score (PS) nearest-neighbor matching strategy. PS was computed based on a comprehensive set of 64 pre-exposure and procedural covariates measured in the 365 days before the ISR procedure, as listed in Supplemental Table 2. These covariates included baseline demographics, comorbidities, cardiovascular risk factors, type of acute coronary syndrome (ACS), category of medical procedures, Charlson Comorbidity Index at the day of the procedure, medication usage, and use of intravascular imaging, all recorded within the year preceding the revascularization event. By convention, a standardized mean difference (SMD) within ± 0.1 was considered to be a good between-group balance.[18]

A Cox proportional hazards model was fitted to the data of the overall cohort. This model included follow-up time, censoring indicators, and the allocated treatment group, with the treatment variable being the sole predictor. Hazard ratios (HRs) and 95% confidence intervals (CIs) were computed accordingly. End points for the matched cohorts were visualized using Kaplan–Meier survival curves. These functions were plotted over a 1-year period. For nonfatal end points, we applied the Aalen-Johansen estimator to account for competing risks.

All statistical analyses were performed using Python version 3.11.6 and R-Studio version 4.1.2.

## Results

**Fig. 1:**
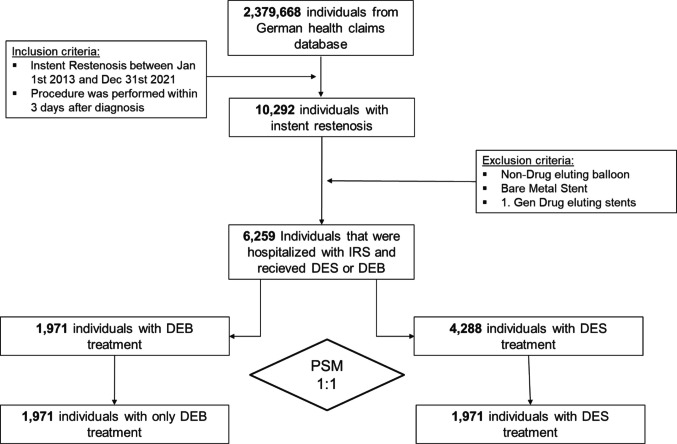
Study overview. Screening, eligibility assessment, and propensity score matching. Specific ICD-10 codes are provided for exclusion criteria. DCB, drug-coated balloon; DES, drug-eluting stent; PSM, propensity score matching

We identified 10,292 individuals with ISR who received invasive treatment by recurrent PCI. Of these, 6259 were treated with either DCB (*n* = 1971) or second-generation DES (*n* = 4288). After applying 1:1 PS matching, a cohort of 3942 patients was included in the inferential analysis (Fig. 1).

Baseline characteristics of individuals before and after PSM are summarized in Table 1. After PS matching, the mean age was 70.3 years for the DCB group and 70.5 years for the second-generation DES group, with women constituting 28% of both populations. Notable differences before propensity score matching included the type of acute coronary syndrome (ACS), with ST-elevation myocardial infarction (STEMI) present in 5.4% of the DCB group (*n* = 107) compared to 16.3% of the DES group (*n* = 697). Additional differences were observed in the history of PCI within the previous year, seen in 43.2% of the DCB group (*n* = 852) compared to 25.8% of the DES group (*n* = 1107). Furthermore, the DES group underwent more frequent intravascular imaging in the unadjusted cohort (*n* = 315, 7.3%) compared to the DCB group (*n* = 82, 4.2%). PS matching effectively adjusted for these imbalances. The median length of follow-up was 1 year for both groups.

**Table 1:** Baseline characteristics before (left) and after (right) propensity score matching. Pre-exposure characteristics measured in the 1 year prior to in-stent restenosis. Plus–minus values are means ± SD. Values are provided as *n* (%)

Feature	DCB (n = 1971)	2. Gen DES (n = 4288)	SMD	DCB (n = 1971)	2. Gen DES (n = 1971)	SMD
Age	70.3 (10.9)	70.1 (11.3)	0.019	70.3 (10.9)	70.5 (10.9)	−0.019
Sex (male)	1414 (71.7%)	3174 (74.0%)	−0.051	1414 (71.7%)	1413 (71.7%)	0.001
Smoking	405 (20.5%)	933 (21.8%)	−0.03	405 (20.5%)	396 (20.1%)	0.011
Alcohol Abuse	96 (4.9%)	181 (4.2%)	0.031	96 (4.9%)	99 (5.0%)	−0.007
Acute Kidney Injury	138 (7.0%)	428 (10.0%)	−0.107	138 (7.0%)	141 (7.2%)	−0.006
CKD Stage 1 or 2	339 (17.2%)	742 (17.3%)	−0.003	339 (17.2%)	338 (17.1%)	0.001
CKD Stage 3, 4, or 5 incl. Dialysis	599 (30.4%)	1208 (28.2%)	0.049	599 (30.4%)	587 (29.8%)	0.013
Diabetes Mellitus	1073 (54.4%)	2266 (52.8%)	0.032	1073 (54.4%)	1071 (54.3%)	0.002
Insulin Use	403.0 (20.4%)	789.0 (18.4%)	0.052	403.0 (20.4%)	393.0 (19.9%)	0.013
Obesity	678 (34.4%)	1452 (33.9%)	0.011	678 (34.4%)	667 (33.8%)	0.012
Hypertension	1907 (96.8%)	4060 (94.7%)	0.102	1907 (96.8%)	1902 (96.5%)	0.014
Hyperlipidemia	1831 (92.9%)	3948 (92.1%)	0.031	1831 (92.9%)	1842 (93.5%)	−0.022
Statin Use	1630 (82.7%)	3203 (74.7%)	0.196	1630 (82.7%)	1617 (82.0%)	0.017
Atrial Fibrillation	340 (17.3%)	690 (16.1%)	0.031	340 (17.3%)	336 (17.0%)	0.005
Old Myocardial Infarction	1072 (54.4%)	2167 (50.5%)	0.077	1072 (54.4%)	1066 (54.1%)	0.006
Congestive Heart Failure	1154 (58.5%)	2511 (58.6%)	0	1154 (58.5%)	1156 (58.7%)	−0.002
Peripheral Artery Disease	442 (22.4%)	861 (20.1%)	0.057	442 (22.4%)	448 (22.7%)	−0.007
PCI last year	852 (43.2%)	1107 (25.8%)	0.372	852 (43.2%)	823 (41.8%)	0.03
STEMI	107 (5.4%)	697 (16.3%)	−0.354	107 (5.4%)	106 (5.4%)	0.002
NSTEMI	398 (20.2%)	1118 (26.1%)	−0.14	398 (20.2%)	400 (20.3%)	−0.003
Unstable Angina Pectoris	419 (21.3%)	674 (15.7%)	0.143	419 (21.3%)	423 (21.5%)	−0.005
One Vessel Coronary Artery Disease	531 (26.9%)	995 (23.2%)	0.086	531 (26.9%)	535 (27.1%)	−0.005
Two Vessel Coronary Artery Disease	762 (38.7%)	1613 (37.6%)	0.021	762 (38.7%)	773 (39.2%)	−0.011
Three Vessel Coronary Artery Disease	1259 (63.9%)	2865 (66.8%)	−0.062	1259 (63.9%)	1274 (64.6%)	−0.016
Left Main Coronary Artery Disease	207 (10.5%)	560 (13.1%)	−0.079	207 (10.5%)	201 (10.2%)	0.01
Intravascular Imaging	82 (4.2%)	315 (7.3%)	−0.137	82 (4.2%)	94 (4.8%)	−0.029
Anticoagulation Use	399 (20.2%)	757 (17.7%)	0.066	399 (20.2%)	403 (20.4%)	−0.005
COPD/Asthma	368 (18.7%)	819 (19.1%)	−0.011	368 (18.7%)	421 (21.4%)	−0.067
Charlson Comorbidity Index	8.7 (4.0)	8.6 (4.0)	0.041	8.7 (4.0)	8.8 (3.8)	−0.009
Medical Procedure Count	9.4 (6.9)	8.7 (6.3)	0.114	9.4 (6.9)	9.4 (6.7)	0
Intensive Care Count	0.3 (0.6)	0.3 (0.6)	−0.115	0.3 (0.6)	0.3 (0.5)	−0.001
Unique Medication Count	12.0 (5.6)	10.5 (5.7)	0.272	12.0 (5.6)	11.9 (5.8)	0.012

**Fig. 2:**
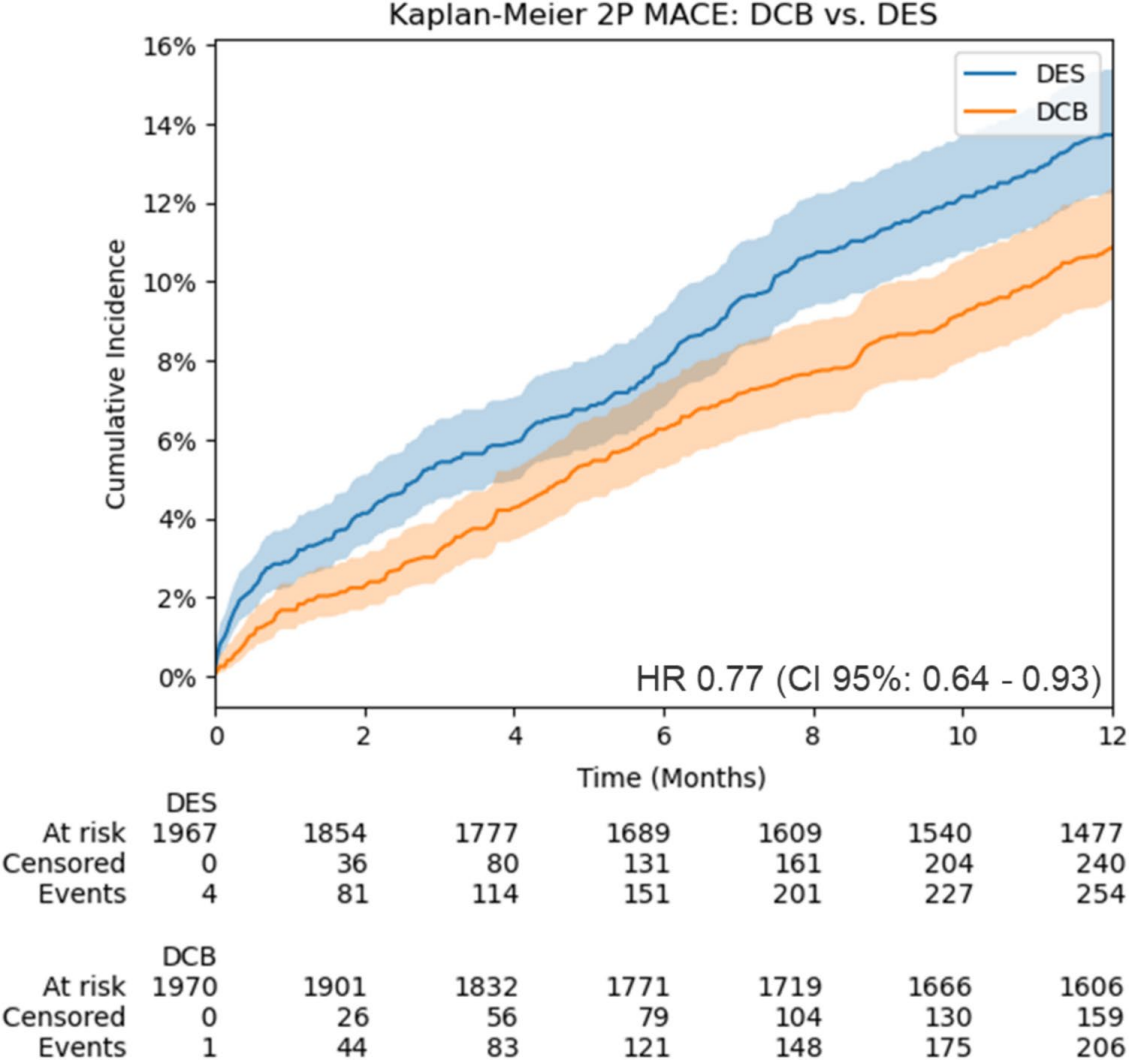
Primary end point at 1 year. DCB vs. DES after propensity score matching: cumulative incidence of the primary end point, the composite of death or myocardial infarction at 1 year after in-stent restenosis. DCB: drug-coated balloon; DES: 2nd-generation drug-eluting stent; HR: hazard ratio

*CKD*: chronic kidney disease; *PCI*: percutaneous coronary intervention; *STEMI*: ST-elevation myocardial infarction;* NSTEMI*: Nnon-ST-Eelevation myocardial infarction; *COPD*: chronic obstructive pulmonary disease;

At the 1-year follow-up, the composite primary end point of all-cause mortality or myocardial infarction (MI) was documented in 10.4% of patients in the DCB group (*n* = 206), versus 12.9% (n = 254) in the DES group (HR, 0.77; 95% CI, 0.64–0.93) (Fig. 2).

**Fig. 3:**
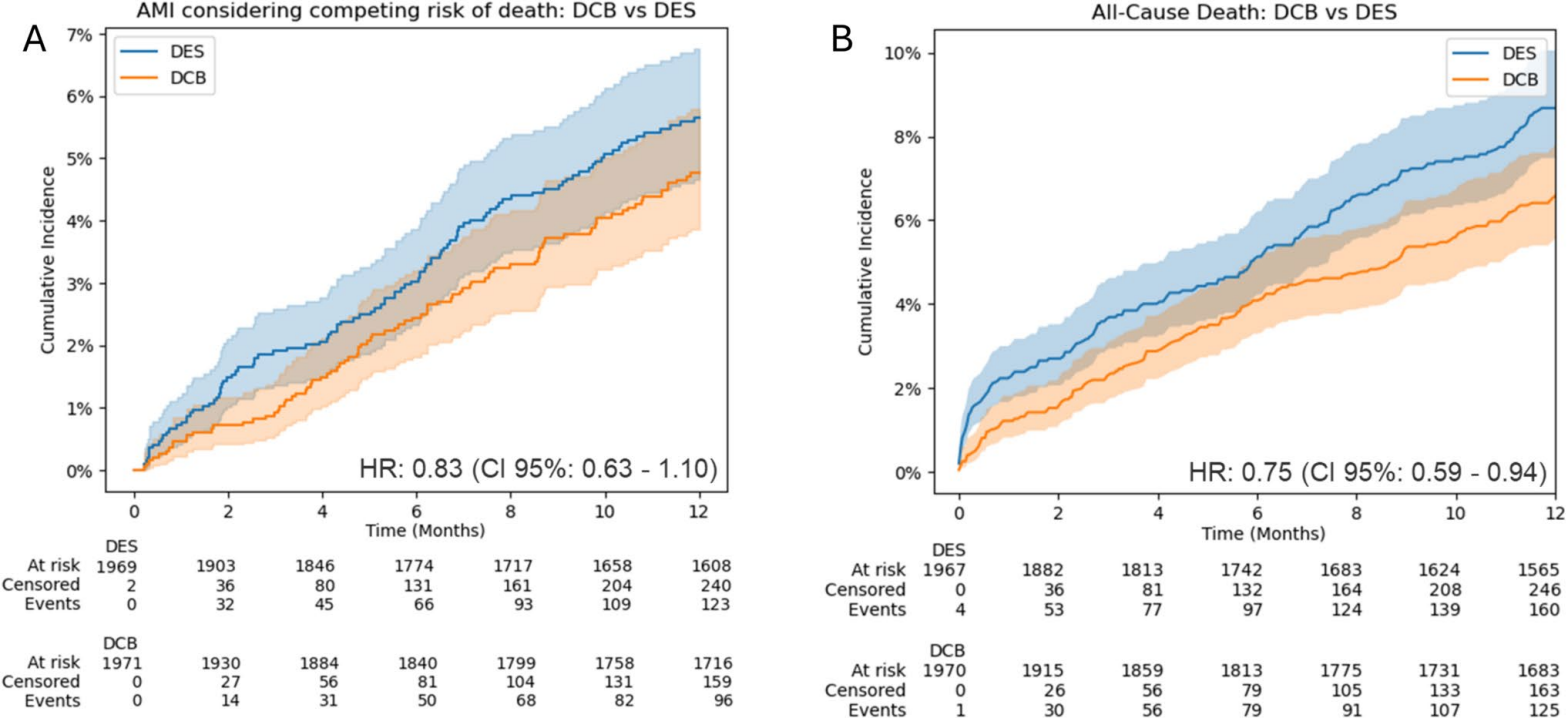
Secondary end points at 1 year: (**A**) all-cause death and (**B**) myocardialinfarction. DCB vs. DES after propensity score matching: incidence of the secondary end points: (**A**) all-cause death and (**B**) acute myocardial infarction at 1 year. Acute myocardial infarction estimation accounted for the competing risk of death using the Aalen–Johansen method. DCB: drug-coated balloon; DES: 2nd-generation drug-eluting stent; HR: hazard ratio; AMI: acute myocardial infarction

All-cause mortality was observed in 6.3% of patients (*n* = 125) treated with DCB, and in 8.1% of patients (*n* = 160) treated with DES (HR, 0.75; 95% CI, 0.59–0.94) (Fig. 3A). The incidence of MI was 4.9% in the DCB cohort (*n* = 96) and 6.2% in the DES cohort (*n* = 123) (HR, 0.83; 95% CI, 0.63–1.10) (Fig. 3B).

**Fig. 4:**
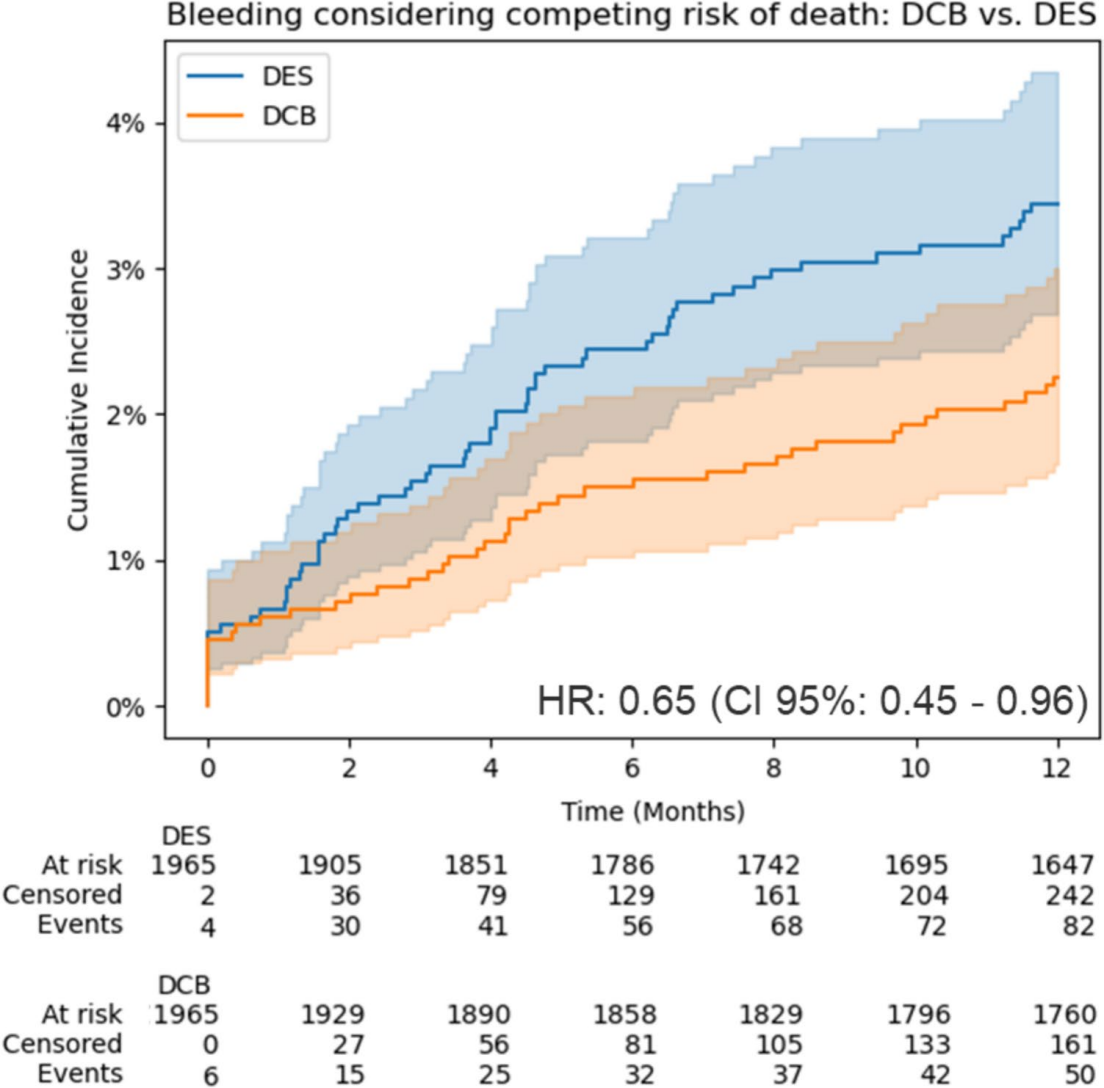
Safety end point at 1 year: DCB vs. DES after propensity score matching: bleeding estimation accounted for the competing risk of death using the Aalen–Johansen method. DCB: drug-coated balloon; DES: 2nd-generation drug-eluting stent; HR: hazard ratio

**Fig. 5:**
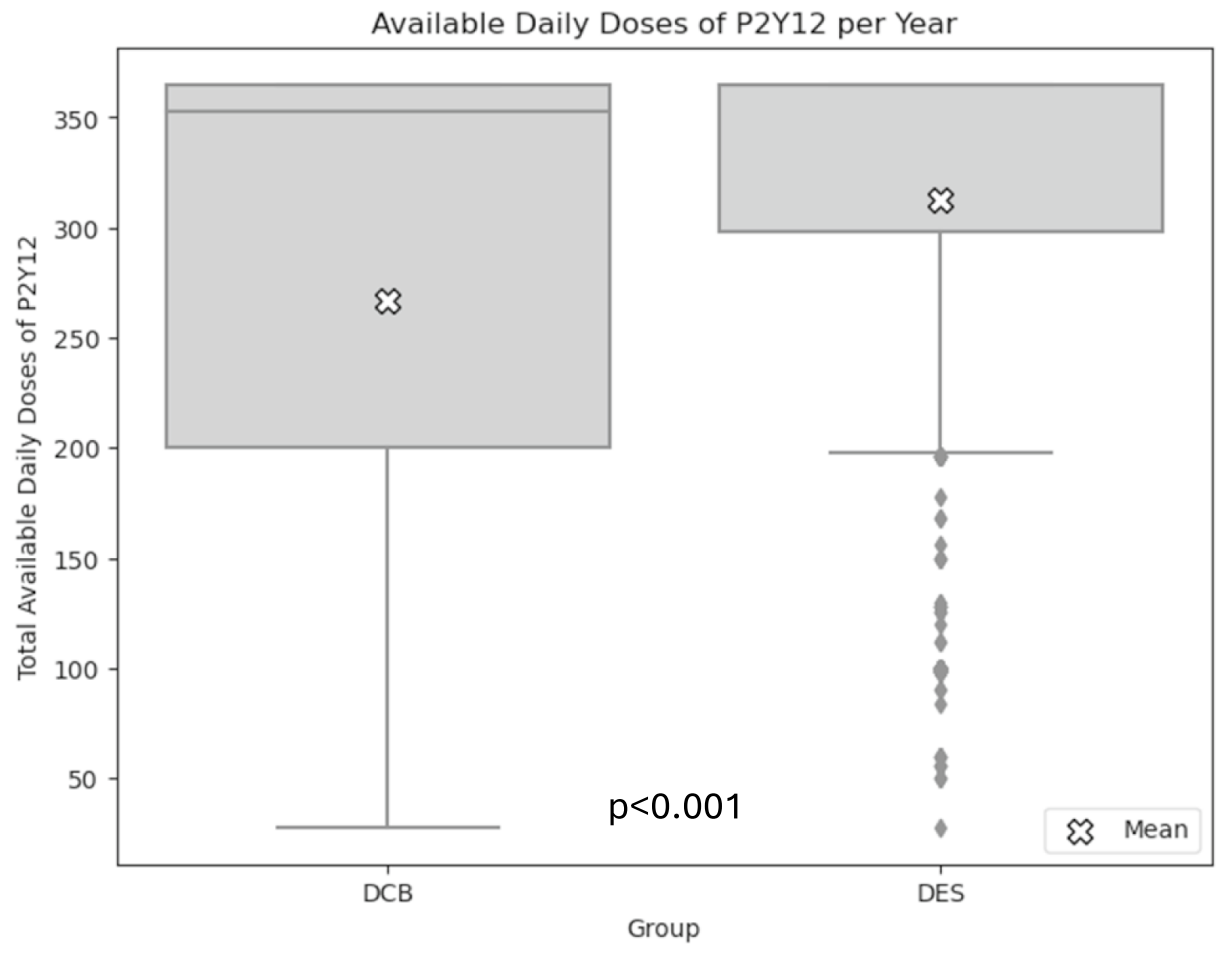
Available daily doses of P2Y12 per year. Comparison of total available daily doses for patients using drug-coated balloons (DCB) versus 2nd-generation drug-eluting stents (DES). Boxplots show median, Q1, and Q3; white crosses denote group means. The cut-off was set at 365 daily doses. DCB: drug-coated balloon; DES: 2nd-generation drug-eluting stent; HR: hazard ratio

Hospitalization for severe bleeding events was significantly higher in the DES group, occurring in 4.2% (n = 82) and 2.5% (*n* = 50) in the DCB group (HR, 0.65; 95% CI, 0.45–0.96) (Fig. 4). While anticoagulation use was balanced due to PS matching, the analysis of the median daily doses per year revealed significantly higher usage of P2Y12 inhibitors in the DES arm compared to the DCB arm. When a maximum of 365 daily doses was reachable, the mean daily doses per year were 266.8 (± 117.5) for the DCB group versus 312.5 (± 85.3) for the DES group (*p* < 0.001) (Fig. 5).

The sensitivity analysis restricted to patients with single-vessel intervention at the time of restenosis, therefore solely the restenosis being treated, confirmed the primary outcome (HR, 0.78; 95% CI, 0.65–0.95) (Supplemental Fig. 1). Secondary outcomes were also confirmed, showing an HR for all-cause mortality of 0.74 (95% CI, 0.59–0.95) and for acute myocardial infarction of 0.89 (95% CI, 0.67–1.21) (Supplemental Fig. 2 A + B). The occurrence of severe bleeding did not differ significantly in the single-vessel intervention group (HR, 0.68; 95% CI, 0.45–1.02) (Supplemental Fig. 3). In the subgroup limited to patients with single-vessel CAD, results were consistent with the primary cohort, with a lower risk of the primary outcome in the DCB group (HR, 0.63; 95% CI, 0.42–0.97) (Supplemental Fig. 4).

No difference in the two negative controls, pneumonia (HR, 0.99; 95% CI, 0.83–1.20) and surgical hip replacement (HR, 0.90; 95% CI, 0.40–2.30) was observed (Supplemental Fig. 5).

In the secondary cohort that retained patients treated with POBA, 6818 patients were included (DES *n* = 4248; DCB *n* = 1947; and POBA *n* = 623). Age and sex distributions were comparable across groups; baseline characteristics are provided in Supplemental Table 3. At 1 year, the composite primary end point of all-cause mortality or myocardial infarction occurred in 118 patients in the POBA group (18.9%), 204 in the DCB group (10.5%), and 614 in the DES group (14.5%) (Supplemental Fig. 6). Relative to POBA, event risk was lower with DES (HR, 0.75; 95% CI, 0.62–0.92) and with DCB (HR, 0.52; 95% CI, 0.41–0.65).

## Discussion

In this study, we report the clinical outcomes of 3942 patients who underwent interventional treatment by recurrent PCI of ISR with either second-generation DES or DCB. The main findings are the following: First, at 1-year follow-up, the incidence of the composite primary end point of all-cause mortality or MI was significantly higher in patients treated with second-generation DES. Second, similar findings were observed regarding all-cause mortality and hospitalization for bleeding, which was substantially higher in the DES group. Third, the 1-year incidence of MI was comparable between both groups.

Various treatment modalities have been used and investigated for the treatment of ISR. However, the outcomes for most of these strategies were disappointing, except for second-generation DES implantation and DCB angioplasty.[19] Until recently, the ESC endorsed both techniques with a class I, level A recommendation.[3] Recent evidence supporting the superiority of DES with respect to TLR led to the revision of those guidelines with recommendations to favor DES over DCB.[6] However, major cardiovascular outcomes did not differ between the two groups, with all-cause death, MI, and target lesion thrombosis indicating a numerical excess in the DES group.[5] Paclitaxel-DCBs are widely used in Europe and can also be assumed to be prevalent in our study cohort. The clinical advantage of DCB implantation lies in its ability to dilate restenosis, deliver antiproliferative drugs locally to inhibit neointimal growth, avoid the placement of an additional stent layer, and the prevention of further decrease in vasomotion of the coronary artery. In contrast, repeat DES implantation provides sustained radial force and has been associated with favorable angiographic outcomes.[19]

Data supporting our findings come from the 10-year results of the ISAR-DESIRE 3 trial.[11] In this study, 402 patients were treated with either plain old balloon angioplasty, DCB angioplasty, or first-generation DES implantation. The long-term follow-up revealed higher rates of all-cause mortality or MI within 5 years in the first-generation DES group compared to the DCB group (HR, 1.97; 95% CI, 1.11–3.52), which was not statistically significant after 10 years. Furthermore, an increased rate of all-cause mortality was noted for the DES group versus the DCB group after 10 years (HR, 1.48; 95% CI, 1.00–2.21).[11]

A large-scale meta-analysis of 10 randomized trials[12] evaluated 1976 patients regarding their outcomes after DES implantation or DCB angioplasty. Although a higher incidence of target lesion revascularization was detected after 3 years for DCB (HR, 1.32; 95% CI, 1.02–1.70), the rates for all-cause mortality (HR, 0.81; 95% CI, 0.53–1.22), and myocardial infarction (HR, 0.95; 95% CI, 0.61–1.48) were comparable between the two, with numerically lower rates favoring DCB.[12] It should be noted that this meta-analysis included BMS-ISR and DES-ISR, and that first- and second-generation DES were allowed in the DES group. RIBS IV (*n* = 309) and RESTORE (*n* = 172) are the only available trials evaluating second-generation DES versus DCB exclusively in DES-ISR.[13, 20] In the RIBS IV trial, although angiographic follow-up results and the 3-year target lesion revascularization rate (HR, 0.33; 95% CI, 0.14–0.79) favored DES over DCB, no differences were observed for all-cause mortality (HR, 1.32; 95% CI, 0.30–5.90) and the occurrence of myocardial infarction (HR, 0.40; 95% CI, 0.08–2.04).[13] DARE and BIOLUX-RCT were based on second-generation DES but included both DES- and BMS-ISR. Both studies demonstrated that DCB was noninferior to DES in terms of angiographic results.[21, 22]

A possible explanation for our findings may be that repeat DES implantation leads to at least two stent layers (or multiple layers in case of recurrent ISR), potentially leading to luminal narrowing, an increased risk of myocardial infarction and stent thrombosis, and consequently, major adverse cardiac events.[14, 23, 24] In the present study, an evident numerical trend favoring DCB over DES for the individual outcome of MI was observed (HR, 0.83; 95% CI, 0.63–1.10), but this effect did not reach the threshold of statistical significance and could only partially explain the mortality reduction.

Finally, the present study shows a reduction of 1-year bleeding events in patients treated with DCB compared with those treated with DES, likely associated with a reduced duration of dual antiplatelet therapy. Indeed, although current guidelines empirically recommend 3–12 months of dual antiplatelet therapy following DCB angioplasty, evidence from routine care indicates that treatment durations are generally shorter.[25] For these reasons, the limited dual antiplatelet therapy requirements of DCB angioplasty should be compared with DES in the setting of patients at high bleeding risk. Ultimately, regardless of the outcomes, neither DCB nor DES should be considered a universal solution for ISR, highlighting the importance of utilizing intravascular imaging to guide the careful selection of an individualized treatment strategy tailored to the specific characteristics of each case.

### Limitations

Several important limitations must be acknowledged. First, the retrospective nature of this study may introduce confounding, though extensive propensity score matching helped achieve good between-group balance. Second, using insurance claims data limited access to angiographic and procedural details such as vessel diameter, lesion morphology, ISR type, prior stent generation, and whether ISR was first-time or recurrent. Consequently, we could not determine whether DCB versus DES selection was influenced by lesion characteristics. Although the exact duration of P2Y12 therapy may not be perfectly captured, large claims databases are widely recognized as reliable for medication use in pharmacoepidemiologic research.[26] The value of detailed intravascular imaging has been demonstrated.[27] Although its findings were unavailable in our data, intravascular imaging use was incorporated as a variable in our PS model. Third, lesion-level outcomes were unavailable, so we could not directly link death or MI to the treated ISR segment, nor determine target lesion revascularization. While this makes precise attribution speculative, our sensitivity analyses were specifically designed to approximate target lesion failure and provide a reasonable surrogate, thereby strengthening confidence in the observed associations.

## Conclusion

This study showed that DCBs were associated with a lower incidence of the composite of all-cause death and myocardial infarction, as well as fewer bleeding events at 1 year in coronary ISR, compared to second-generation DES. Our results add real-world evidence supporting the use of DCBs in coronary ISR. However, these findings should not replace the need for large-scale randomized controlled trials with robust clinical end points, as exemplified in this study.

## Supplementary Information

Below is the link to the electronic supplementary material.

ESM1 (DOCX. 160 KB)

## Data Availability

The data supporting the results presented are available from the corresponding author upon reasonable request

## Abbreviations

DCB: Drug-coated balloon
DES: Drug-eluting stents
POBA: Plain old balloon angioplasty
ISR: In-stent restenosis
PCI: Percutaneous coronary intervention
MI: Myocardial infarction
STEMI: ST-segment elevation myocardial infarction
OBSERVABLE: Observational Bavarian Health Insurance Registry
ICD: International Classification of Diseases
ATC: Anatomical Therapeutic Chemical
PZN: Pharmaceutical central number
PSM: Propensity score matching
