# Supplementary material for: Clinical outcomes of drug-coated balloon vs. second-generation drug-eluting stent for coronary in-stent restenosis
Source: Clin Res Cardiol. 2025 Nov 25;115(1):121–31. doi: 10.1007/s00392-025-02781-7 (PMC12783194; doi:10.1007/s00392-025-02781-7)

**Comparative Outcomes of Drug-Coated Balloon Angioplasty versus Second-Generation DES for ISR: A Real-World Analysis**

Krefting J. et al.

**Supplemental Tables:**

**Supplemental Table 1 – Diagnoses, procedures and medication list**

**Supplemental Table 2 – Preexposure variables for propensity score matching (PSM)**

**Supplemental Table 3 - Baseline characteristics of secondary cohort including Plain Old Balloon Angioplasty**

**Supplemental Figures:**

**Supplemental Figure 1 – Sensitivity analysis: Primary end point 1 year following single vessel intervention**

**Supplemental Figure 2 – Sensitivity analysis: Secondary end points 1 year following single vessel intervention**

**Supplemental Figure 3 – Sensitivity analysis: Bleeding 1 year following single vessel intervention**

**Supplemental Figure 4 – Sensitivity analysis: Primary endpoint at 1 year following In-stent Restenosis of patients with single vessel coronary artery disease**

**Supplemental Figure 5 – Negative Control: Incidence of pneumonia and total hip replacement at 1 year following single vessel interventions**

**Supplemental Figure 6 – Secondary Cohort including Plain Old Balloon Angioplasty: Primary endpoint at 1 year following In-stent Restenosis**

**Supplemental Table 1 – Diagnoses, procedures and medication list.** Provided are A) International Classification of Diseases 10th Revision (ICD-10-GM), B) German procedure classification system (OPS) and C) Anatomical Therapeutic Chemical classification system (ATC) codes used in this study.

| **Diagnosis / Procedure / Medication** | **Code** |
| --- | --- |
| **A) *International Classification of Diseases 10th Revision (ICD-10-GM)*** | |
| Aclohol Abuse | F10 |
| Acute Kidney Failure | N17 |
| Acute Myocardial Infarction | I21, I22 |
| Acute Stroke | I63, I64 |
| Adipositas | E66 |
| Aortic Atherosclerosis | I70.0 |
| Arterial Embolism Or Thrombosis | I74 |
| Arterial Hypertension | I10, I15 |
| Asthma | J45 |
| Atherosclerosis | I70 |
| Atherosclerotic Heart Disease | I25.1 |
| Atrial Fibrilation and Flutter | I48 |
| Arterial Embolism Or Thrombosis | I74 |
| AV Block Grade II and III | I44.1, I44.2 |
| Bleeding | I60, I61, I62, K92.0, K92.1, K92.2 |
| Carotid stenosis | I65.2 |
| Chronic Heart Failure | I50 |
| Chronic Ischemic Heart Disease | I25 |
| Chronic Ischemic Heart Disease with at least 2 vessel disease | I25.12, I25.13 |
| Chronic Kidney Disease | N18* |
| Chronic Kidney Failure Stage 1 And 2 | N18.1, N18.2 |
| Chronic Kidney Failure Stage 3, 4 And 5 | N18.3, N18.4, N18.5 |
| Chronic Renal Insufficiency Requiring Dialysis | N18.5 |
| COPD | J44 |
| Diabetes Mellitus | E10, E11, E12, E13, E14 |
| Diabetes with Endorgan Involvement | E11.2, E11.3, E11.4, E11.5, E10.2, E10.3, E10.4, E10.5 |
| Dyslipidemia | E78 |
| Hypertensive Heart and/or Kidney Disease | I11, I12, I13 |
| Instent Restenosis | I25.16 |
| Intracranial Hemorrhage | I61 |
| Intracranial Arteriovenous Malformation or Intracranial Aneurysm | I67.1 |
| Malignant Hypertension | I10.1 |
| Nicotine Abuse | F17 |
| Non-ST Elevation Myocardial Infarction | I21.4 |
| Old Myocardial Infarction | I25.2 |
| Other Cardiac Arrhythmias | I49 |
| Other Chronic Kidney Disease | N18.8, N18.9 |
| Peripheral Artery Disease | I70.2 |
| Pneumonia | J13, J14, J15, J16 |
| Pulmonary Embolism | I26 |
| Renal Atherosclerosis | I70.1 |
| Sick Sinus Syndrome | I49.5 |
| ST- Segment Elevation Myocardial Infarction | I21.0, I21.1, I21.2, I21.3, I22.0, I22.1, I22.8 |
| Stable Angina Pectoris | I20.8 |
| Stent Thrombosis | I25.16 |
| Transient Ischemic Attack | G45 |
| Unstable Angina Pectoris | I20.0 |
| Venous Thrombosis | I80, I81, I82 |
| Pneumonia | J13 – J18 |
| ***B) German procedure classification system (OPS)*** | |
| 2. Generation Drug Eluting Stent | 8-83b.00, 8-83b.01, 8-83b.07, 8‑83b.08, 8-83b.0b, 8-83b.0c, 8‑83b.0f, 8-83B.02 |
|  | |
| Coronary Artery Bypass Graft Surgery | 5-36* |
| Complex Treatment | 8-98* |
| Diagnostic Cardiac Catheterization | 1-275* |
| Drug Coated Balloon | 8-837.00, 8-837.01, 8-83b.b6, 8‑83b.b7, 8-83b.b8, 8-83b.b9 |
| (Percutaneous) Transluminal Stenting | 8-837*  Excl.: 8-837.7*, 8-837.8, 8-837.9, 8-837.a*, 8-837.b*, 8-837.c*, 8-837.d*, 8-837.e, 8-837.f, 8-837.g, 8-837.h, 8-837.j, 8-837.s* |
| ***C) Anatomical Therapeutic Chemical classification system (ATC)*** | |
| ACE Inhibitors | C09A, C09BA01, C09BA02, C09BA03, C09BA04, C09BA05, C09BA06, C09BA07, C09BA08, C09BA09, C09BA12, C09BA13, C09BA15, C09BA21, C09BA22, C09BA23, C09BA25, C09BA26, C09BA27, C09BA28, C09BA29, C09BA33, C09BA35, C09BA54, C09BA55, C09BB |
| Aldosteron Antagonists | C03DA, C03EC, C03ED |
| Angiotensin II Receptor Blockers | C09CA, C09DA01, C09DA02, C09DA03, C09DA04, C09DA06, C09DA07, C09DA08, C09DA09, C09DA10, C09DA21, C09DA22, C09DA23, C09DA24, C09DA26, C09DA27, C09DA28, C09DB |
| Angiogenesis Inhibitors | C09DX |
|  |  |
| Antiarrhythmics | C01B, C01AA |
| Antihypertensives | C02 |
| Beta Blocking Agents | C07A, C07B, C07C, C07D, C07E, C07FB |
| Calcium Channel Blockers | C07FB, C08, C09BB, C09DB |
| Drugs for obstructive airway diseases | R03 |
| Heparin | B01AB |
| Loop Diuretics | C03C, C03EB, C03ED, C07C, C07D, C09BA55 |
| Thiazides | C03A, C03EA, C07B, C07D, C09BA21, C09BA22, C09BA23, C09BA25, C09BA26, C09BA27, C09BA28, C09BA29, C09BA33, C09BA35, C09BA54, C09DA21, C09DA22, C09DA23, C09DA24, C09DA26, C09DA27, C09DA28 |
| Other Diuretics | C03B, C03DB, C03EA, C03EB, C03EC, C03X, C09BA01, C09BA02, C09BA03, C09BA04, C09BA05, C09BA06, C09BA07, C09BA08, C09BA09, C09BA12, C09BA13, C09BA15, C09DA01, C09DA02, C09DA03, C09DA04, C09DA06, C09DA07, C09DA08, C09DA09, C09DA10 |
| Nitrates | C01DA |
| NSAIDs | M01A, M01BA, N02BA |
| Non-Opioid Analgetica | N02BB, N02BE, N02BG |
| Opioids | N02A |
| Oral Immunosuppressants | L04 |
| Statins | C10AA |
| Other Lipid Modifying Agents | C10AB, C10AC, C10AD, C10AX |

**Supplemental Table 2 – Preexposure variables for propensity score matching (PSM).** Provided are the individual variables for the applied propensity score matching. AKI: Acute Kidney Injury; CABG: Coronary Artery Bypass Graft; CCI: Charlson Comorbidity Index; CKD: Chronic Kidney Disease; CHF: Congestive Heart Failure; COPD: Chronic Obstructive Pulmonary Disease; CVD: Cardiovascular Disease; MI: Myocardial Infarction; NSTEMI: Non-ST-Elevation Myocardial Infarction; PAD: Peripheral Artery Disease; PCI: Percutaneous Coronary Intervention; ARB: Angiotensin receptor blockers; SGLT2: Sodium-Glucose Cotransporter-2; STEMI: ST-Elevation Myocardial Infarction

|  | **Preexposure Variables for PSM** |
| --- | --- |
| 1 | Age |
| 2 | Sex |
| 3 | Number of vessels being intervened |
| 4 | Smoking |
| 5 | Alcohol Abuse |
| 6 | Hypertensive Heart Kidney Disease |
| 7 | AKI |
| 8 | CKD-Stage 1, 2 |
| 9 | CKD-Stage 3, 4, 5 or Dialysis |
| 10 | CKD Not Further Described |
| 11 | Diabetes |
| 12 | Diabetes With End Organ Involvement |
| 13 | Obesity |
| 14 | Hypertension |
| 15 | Malignant Hypertension |
| 16 | Hyperlipidemia |
| 17 | Atrial Fibrillation |
| 18 | Other Cardiac Arrhythmias |
| 19 | Stable Angina |
| 20 | Prior MI |
| 21 | CHF |
| 22 | PAD |
| 23 | Arterial Embolism Thrombosis |
| 24 | Thrombosis |
| 25 | Lung Embolism |
| 26 | Pneumonia |
| 27 | COPD |
| 28 | Asthma |
| 29 | History of PCI |
| 30 | History of CABG |
| 31 | Medical Procedure Count |
| 32 | Intensive Care Count |
| 33 | STEMI |
| 34 | NSTEMI |
| 35 | Unstable Angina Pectoris |
| 36 | One Vessel CVD |
| 37 | Two Vessel CVD |
| 38 | Three Vessel CVD |
| 39 | Left Main CVD |
| 40 | Unique Medication Count |
| 41 | ACE-Inhibitors |
| 42 | ARB |
| 43 | Beta Blockers |
| 44 | Calcium Channel Blockers |
| 45 | Insulin |
| 46 | SGLT2 Inhibitors |
| 47 | Other Antidiabetic Meds |
| 48 | Antiplatelet Agents |
| 49 | Heparin |
| 50 | Anticoagulants |
| 51 | NSAIDs |
| 52 | Non Opioid Analgesics |
| 53 | Opioids |
| 54 | Antiarrhythmic drugs |
| 55 | Antihypertensives |
| 56 | Loop Diuretics |
| 57 | Thiazide Diuretics |
| 58 | Other Diuretics |
| 59 | Aldosterone Antagonists |
| 60 | Statins |
| 61 | Other Lipid Modifying Agents |
| 62 | COPD/ Asthma |
| 63 | CCI at date of Intervention |
| 64 | Use of intravascular imaging |

**Supplemental Table 3 - Baseline characteristics of secondary cohort including Plain Old Balloon Angioplasty.** Preexposure characteristics measured in the 1 year prior to In-stent Restenosis. Plus-minus values are means ±SD. Values are provided as n (%). POBA: Plain Old Balloon Angioplasty; CKD: Chronic Kidney Disease; PCI: Percutaneous Coronary Intervention; STEMI: ST-Elevation Myocardial Infarction; NSTEMI: Non-ST-Elevation Myocardial Infarction; COPD: Chronic Obstructive Pulmonary Disease.

| **Feature** | **2.Gen DES**  **(n=4248)** | **DCB**  **(n=1947)** | **POBA**  **(n=623)** | **SMD DES vs DCB** | **SMD DES vs POBA** | **SMD DCB vs POBA** |
| --- | --- | --- | --- | --- | --- | --- |
| **Age** | 70.0 (11.3) | 70.3 (10.8) | 69.9 (11.1) | 0.027 | -0.011 | -0.039 |
| **Sex (male)** | 3145 (74.0%) | 1394 (71.6%) | 464 (74.5%) | -0.055 | 0.010 | 0.065 |
| **Smoking** | 928 (21.8%) | 401 (20.6%) | 123 (19.7%) | -0.031 | -0.052 | -0.021 |
| **Alcohol Abuse** | 179 (4.2%) | 93 (4.8%) | 46 (7.4%) | 0.027 | 0.136 | 0.109 |
| **Acute Kidney Injury** | 423 (10.0%) | 137 (7.0%) | 60 (9.6%) | -0.105 | -0.011 | 0.094 |
| **CKD Stage 1 or 2** | 725 (17.1%) | 332 (17.1%) | 106 (17.0%) | -0.000 | -0.001 | -0.001 |
| **CKD Stage 3, 4, or 5 incl. Dialysis** | 1195 (28.1%) | 590 (30.3%) | 183 (29.4%) | 0.048 | 0.027 | -0.020 |
| **Diabetes Mellitus** | 2243 (52.8%) | 1059 (54.4%) | 345 (55.4%) | 0.032 | 0.052 | 0.020 |
| **Insulin Use** | 774 (18.2%) | 398 (20.4%) | 125 (20.1%) | 0.056 | 0.047 | -0.009 |
| **Obesity** | 1438 (33.9%) | 668 (34.3%) | 230 (36.9%) | 0.010 | 0.064 | 0.054 |
| **Hypertension** | 4020 (94.6%) | 1883 (96.7%) | 595 (95.5%) | 0.102 | 0.040 | -0.062 |
| **Hyperlipidemia** | 3909 (92.0%) | 1811 (93.0%) | 560 (89.9%) | 0.038 | -0.074 | -0.112 |
| **Statin Use** | 3168 (74.6%) | 1610 (82.7%) | 488 (78.3%) | 0.199 | 0.089 | -0.110 |
| **Atrial Fibrillation** | 679 (16.0%) | 335 (17.2%) | 123 (19.7%) | 0.033 | 0.098 | 0.065 |
| **Old Myocardial Infarction** | 2136 (50.3%) | 1054 (54.1%) | 301 (48.3%) | 0.077 | -0.039 | -0.117 |
| **Congestive Heart Failure** | 2485 (58.5%) | 1139 (58.5%) | 385 (61.8%) | 0.000 | 0.067 | 0.067 |
| **Peripheral Artery Disease** | 846 (19.9%) | 437 (22.4%) | 132 (21.2%) | 0.062 | 0.031 | -0.030 |
| **PCI last year** | 1081 (25.4%) | 838 (43.0%) | 298 (47.8%) | 0.377 | 0.477 | 0.096 |
| **STEMI** | 696 (16.4%) | 103 (5.3%) | 117 (18.8%) | -0.363 | 0.063 | 0.424 |
| **NSTEMI** | 1113 (26.2%) | 395 (20.3%) | 129 (20.7%) | -0.140 | -0.130 | 0.010 |
| **Unstable Angina Pectoris** | 663 (15.6%) | 413 (21.2%) | 80 (12.8%) | 0.145 | -0.079 | -0.224 |
| **One Vessel Coronary Artery Disease** | 982 (23.1%) | 524 (26.9%) | 192 (30.8%) | 0.088 | 0.174 | 0.086 |
| **Two Vessel Coronary Artery Disease** | 1591 (37.5%) | 750 (38.5%) | 253 (40.6%) | 0.022 | 0.065 | 0.043 |
| **Three Vessel Coronary Artery Disease** | 2837 (66.8%) | 1242 (63.8%) | 369 (59.2%) | -0.063 | -0.157 | -0.094 |
| **Left Main Coronary Artery Disease** | 553 (13.0%) | 204 (10.5%) | 71 (11.4%) | -0.079 | -0.050 | 0.029 |
| **Intravascular Imaging** | 311 (7.3%) | 82 (4.2%) | 43 (6.9%) | -0.134 | -0.016 | 0.118 |
| **Anticoagulation Use** | 747 (17.6%) | 396 (20.3%) | 142 (22.8%) | 0.070 | 0.130 | 0.060 |
| **COPD** | 906 (21.3%) | 425 (21.8%) | 136 (21.8%) | 0.012 | 0.012 | 0.000 |
| **Asthma** | 413 (9.7%) | 192 (9.9%) | 55 (8.8%) | 0.005 | -0.031 | -0.035 |
| **Charlson Comorbidity Index** | 8.6 (4.0) | 8.7 (4.0) | 9.0 (3.8) | 0.047 | 0.102 | 0.053 |
| **Medical Procedure Count** | 8.6 (6.3) | 9.4 (6.9) | 9.9 (7.2) | 0.114 | 0.185 | 0.071 |
| **Intensive Care Count** | 0.3 (0.6) | 0.3 (0.6) | 0.4 (0.7) | -0.118 | 0.123 | 0.237 |
| **Unique Medication Count** | 10.4 (5.7) | 12.0 (5.6) | 11.2 (5.7) | 0.274 | 0.134 | -0.139 |
| **Charlson Comorbidity Index** | 906 (21.3%) | 425 (21.8%) | 136 (21.8%) | 0.012 | 0.012 | 0.000 |
| **Medical Procedure Count** | 413 (9.7%) | 192 (9.9%) | 55 (8.8%) | 0.005 | -0.031 | -0.035 |

**Supplemental Figure 1 –** **Sensitivity analysis: Primary endpoint at 1 year following single vessel interventions.** The Kaplan-Meier curves show the cumulative incidence of the primary outcome of only single vessel interventions, the composite of death or myocardial infarction at 1 year. DCB: Drug coated balloon; DES: Drug eluting stent, HR: Hazard Ratio.


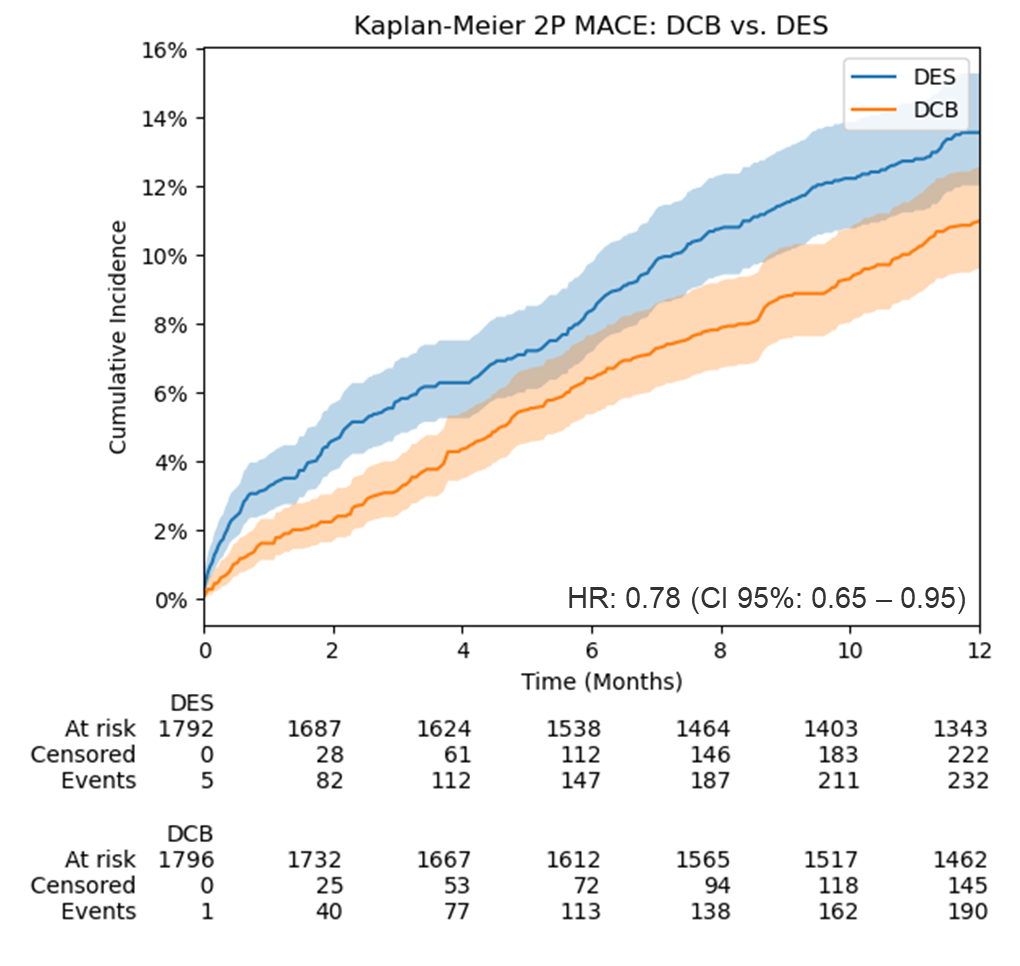


**Supplemental Figure 2 – Sensitivity analysis Secondary end points at 1 year following single vessel interventions. A) All-cause death and B) Acute myocardial infarction**. Acute myocardial infarction was accounted for the competing risk of death using the AalenJohansenFitter. DCB: Drug coated balloon; DES: 2. Generation drug eluting stent, HR: Hazard Ratio.

A

B

**
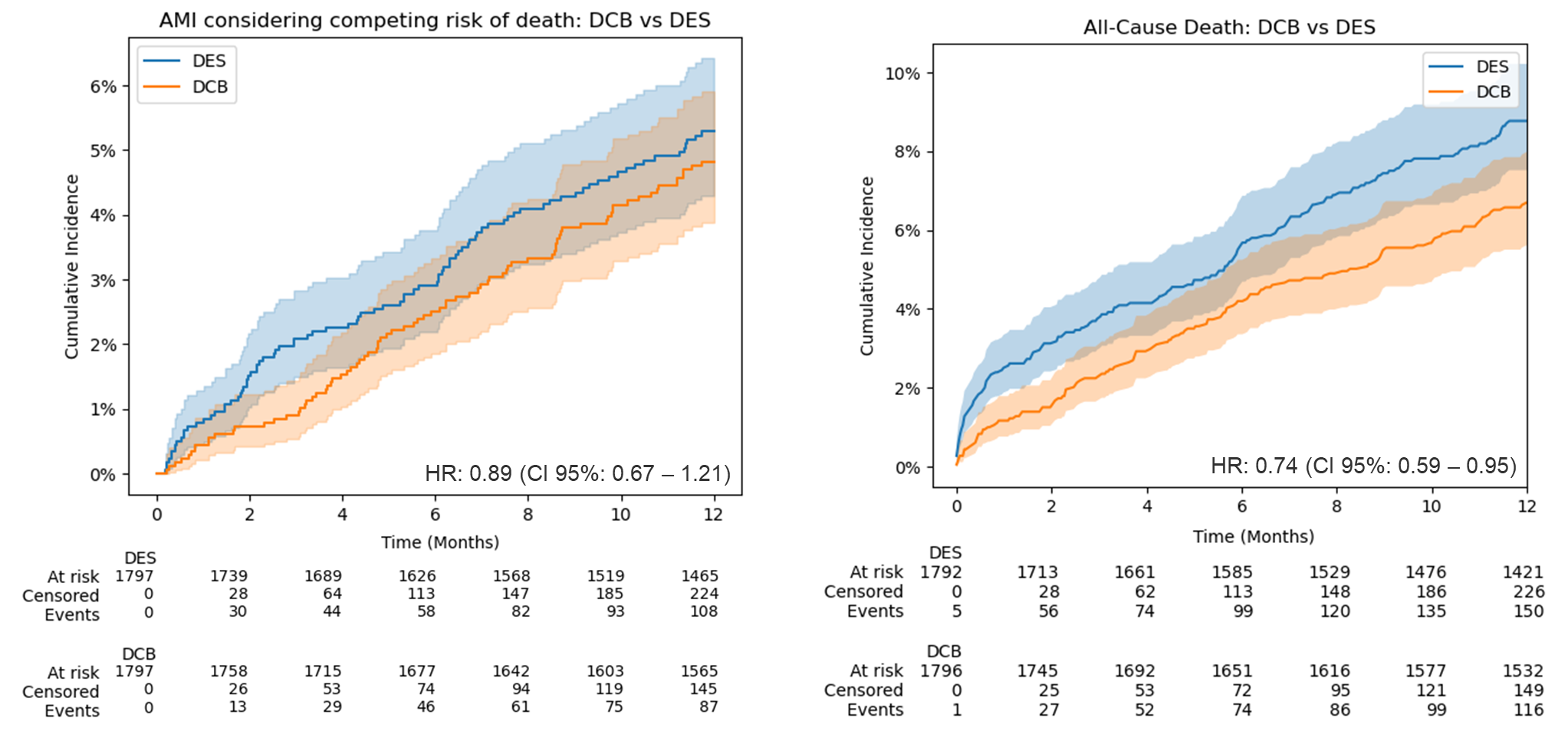
**

**Supplemental Figure 3 – Bleeding at 1 year following single vessel interventions:** DCB vs. DES after Propensity Score Matching: Bleeding estimation accounted for the competing risk of death using the Aalen-Johansen method. DCB: Drug-coated balloon; DES: Drug-eluting stent. HR: Hazard Ratio.


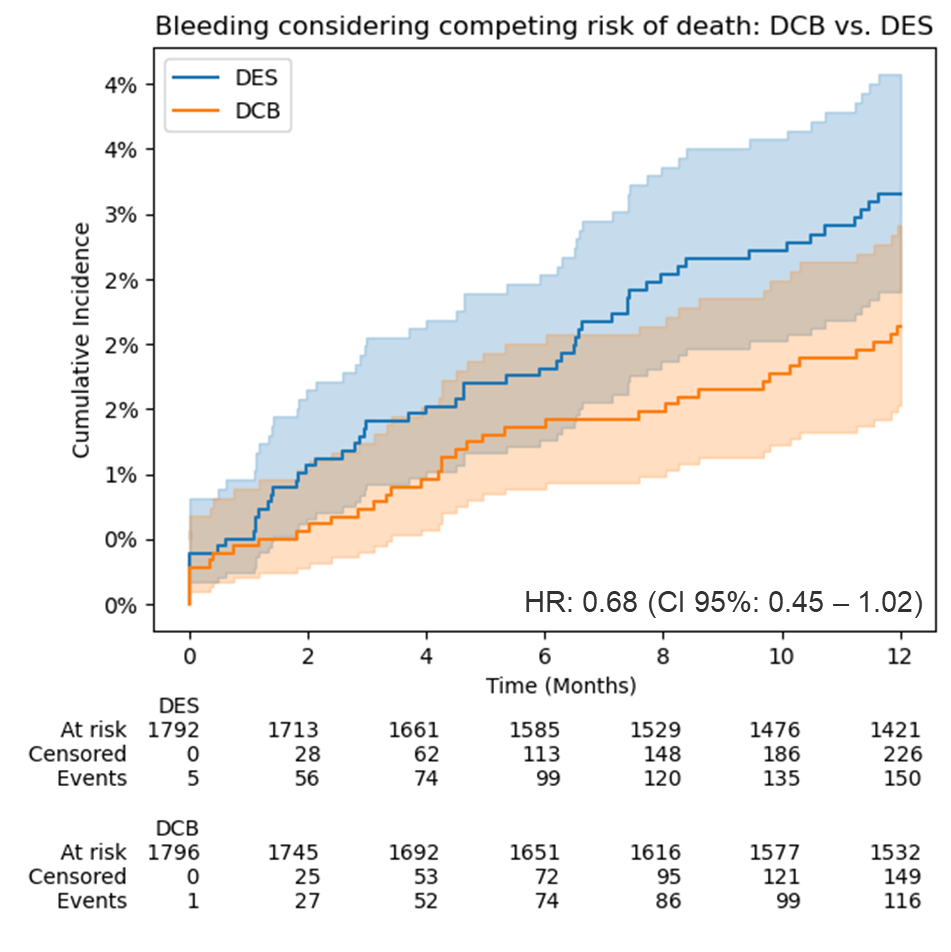


**Supplemental Figure 4 – Sensitivity analysis: Primary endpoint at 1 year following In-stent Restenosis of patients with single vessel coronary artery disease.** The Kaplan-Meier curves show the cumulative incidence of the primary outcome of only patients with one vessel coronary artery disease, the composite of death or myocardial infarction at 1 year. DCB: Drug coated balloon; DES: Drug eluting stent, HR: Hazard Ratio.


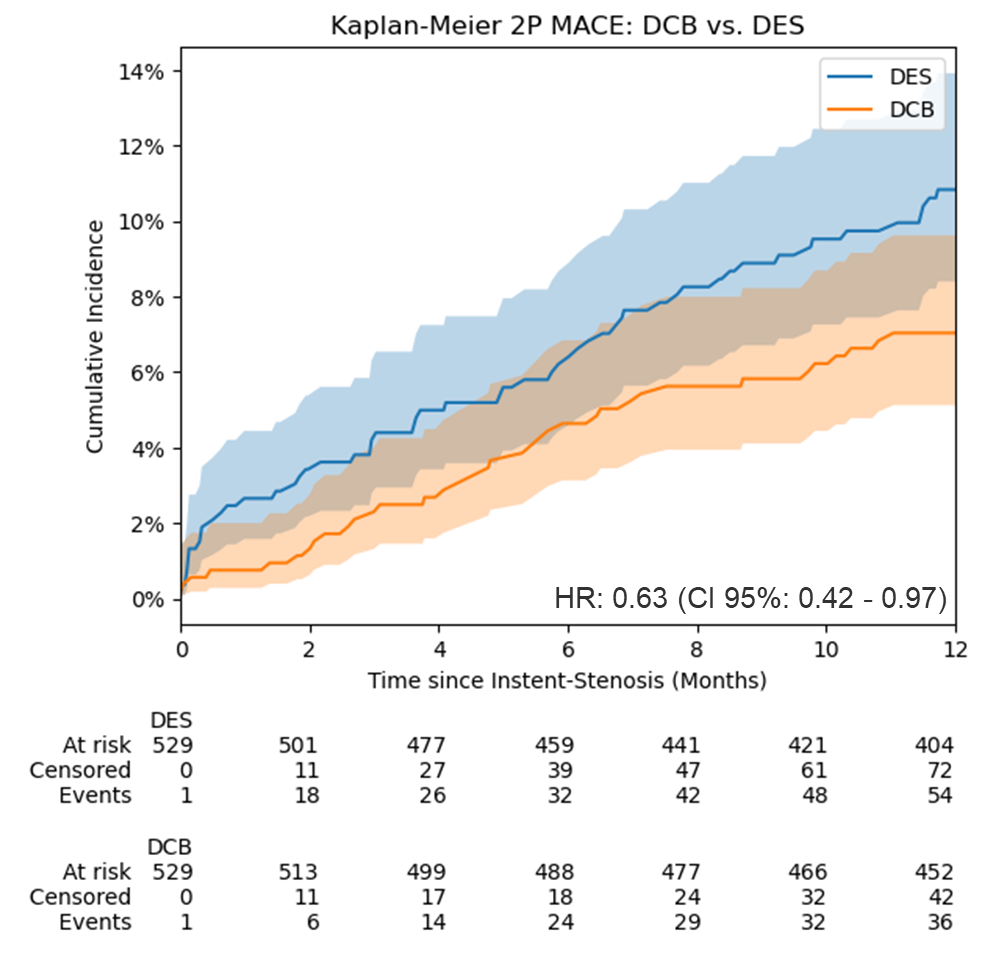


**Supplemental Figure 5 – Negative Control: Incidence of pneumonia and total hip replacement at 1 year following single vessel interventions.** The Kaplan-Meier curves show the cumulative incidence of pneumonia at 1 year. DCB: Drug coated balloon; DES: Drug eluting stent, HR: Hazard Ratio.

**
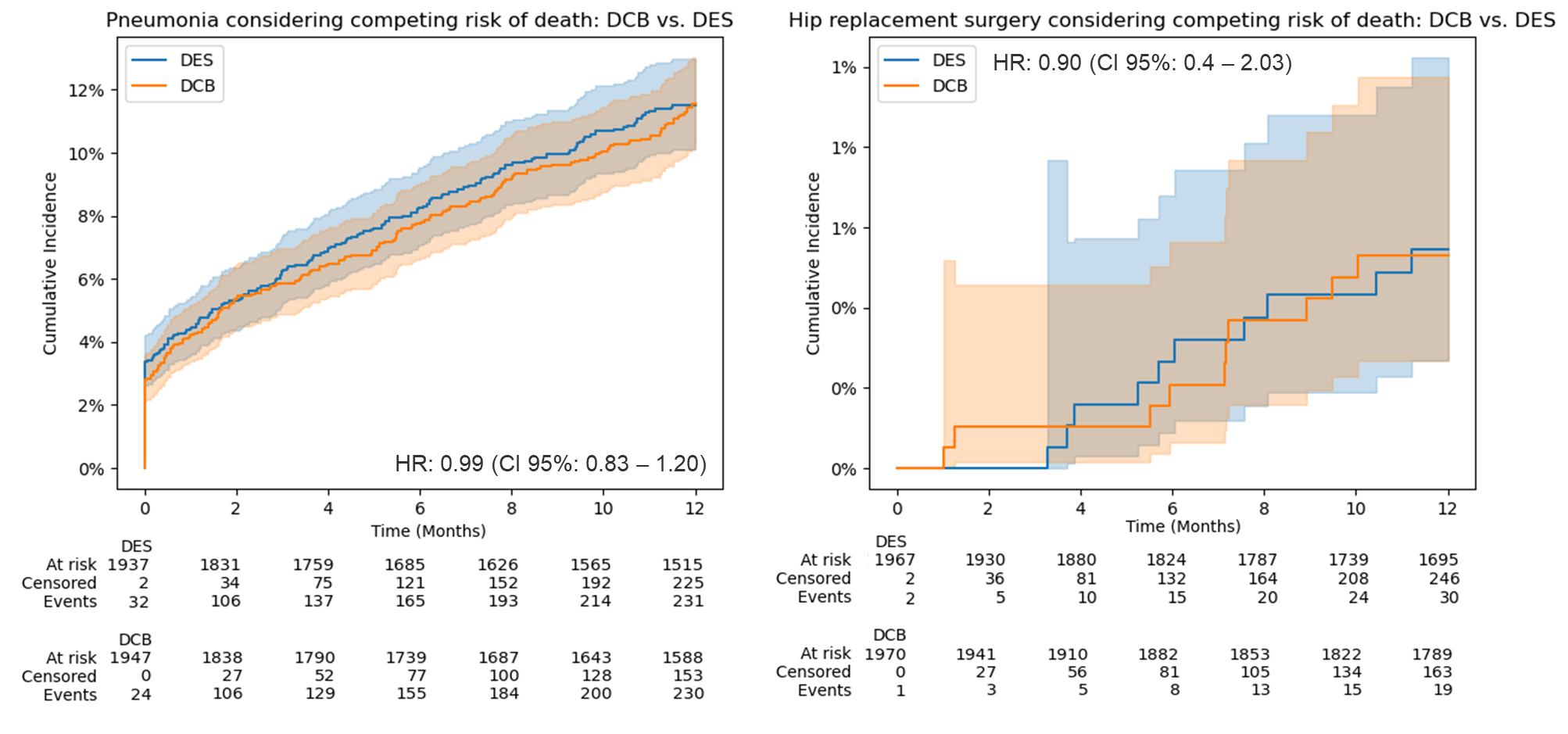
**

**Supplemental Figure 6 – Secondary Cohort including Plain Old Balloon Angioplasty: Primary endpoint at 1 year following Instent Restenosis.** The Kaplan-Meier curves show the cumulative incidence of the primary outcome the composite of death or myocardial infarction of POBA, DCB and second-generation DES at 1 year. POBA: Plain Old Balloon Angioplasty, DCB: Drug coated balloon; DES: Drug eluting stent, HR: Hazard Ratio.


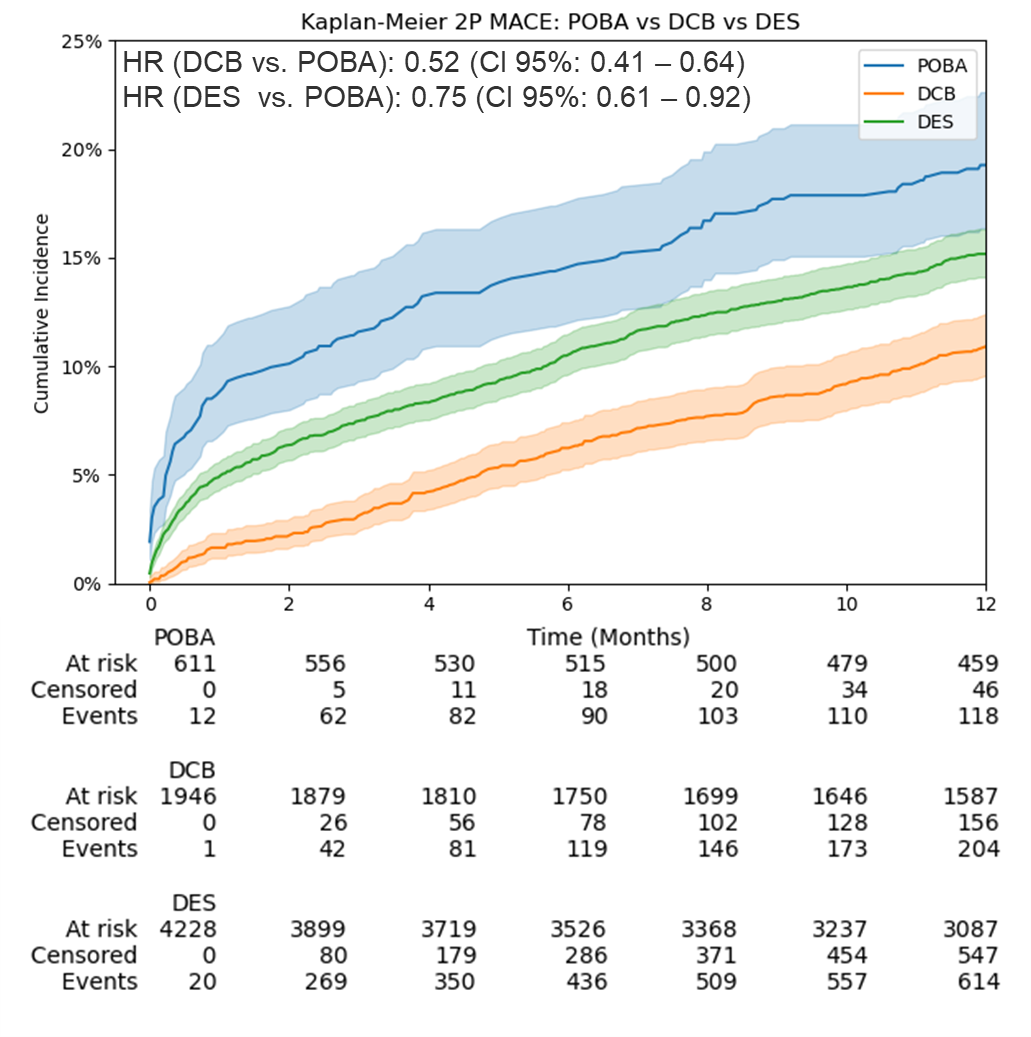

Supplement: Supplementary file 1 — (DOCX. 160 KB) [file 392_2025_2781_MOESM1_ESM.docx]
